# Precision approach to diabetic peripheral neuropathy: modified nerve decompression surgery and MNGF treatment

**DOI:** 10.3389/fmed.2025.1676532

**Published:** 2025-12-19

**Authors:** Yanji Zhang, Hehua Song, Yang Jian, Silang Cai, Chengliang Deng, Zairong Wei

**Affiliations:** 1 Department of Burns and Plastic Surgery, Affiliated Hospital of Zunyi Medical University, Zunyi, Guizhou, China; 2 Department of Medical Education, Kweichow Moutai Hospital, Zunyi, Guizhou, China; 3 The 2011 Collaborative Innovation Center of Tissue Damage Repair and Regeneration Medicine, Affiliated Hospital of Zunyi Medical University, Zunyi, China; 4 The Collaborative Innovation Center of Tissue Damage Repair and Regeneration Medicine, Zunyi Medical University, Zunyi, China

**Keywords:** nerve decompression, diabetic peripheral neuropathy, neuropathic pain, randomized controlled trial, visual analog score

## Abstract

**Background:**

Diabetic peripheral neuropathy (DPN) is a common and debilitating complication of diabetes, often resulting in pain, sensory loss, and lower limb ulcers. While traditional nerve decompression (ND) surgery can alleviate symptoms, it is associated with several limitations, including excessive surgical trauma, postoperative fibrosis, and limited efficacy in advanced cases. To address these challenges, this study aimed to precisely target anatomical sites of peripheral nerve compression identified through cadaveric dissection, perform selective decompression to minimize nerve injury, and investigate the therapeutic potential of combining modified ND with murine nerve growth factor (MNGF) to enhance clinical outcomes.

**Methods:**

In this assessor-blinded, three-arm exploratory randomized controlled trial, 42 patients with confirmed lower limb DPN were randomly assigned to one of three groups: traditional ND (*n* = 12), modified ND (*n* = 16), or modified ND combined with MNGF therapy (*n* = 14). Modified ND involved selective decompression of tendon-like structures without extensive epineurial dissection. Clinical outcomes were assessed preoperatively and at 4 and 12 weeks postoperatively using motor nerve conduction velocity (MNCV), Toronto Clinical Scoring System (TCSS), visual analog scale (VAS), two-point discrimination (2-PD), 10 g monofilament test, and ultrasound-based nerve cross-sectional area (CSA).

**Results:**

All groups showed significant improvements in MNCV, TCSS, VAS, 2-PD, and sensory function after treatment (*p* < 0.05). The modified ND + MNGF group demonstrated the greatest enhancements in nerve conduction and clinical scores, with significant intergroup differences compared to the traditional ND group (*p* < 0.05). Additionally, the modified ND technique reduced surgical trauma and postoperative complications. No severe adverse events were reported.

**Conclusion:**

Modified ND surgery, particularly when combined with MNGF administration, may offer a safe and minimally invasive treatment option for lower-limb DPN. Further validation in larger, multicenter trials is warranted.

## 1 Introduction

With the increasing prevalence of diabetes, its associated complications, particularly DPN, have become a significant global public health challenge (1, 2). DPN not only leads to sensory loss and motor dysfunction but also significantly impairs patients’ quality of life. In severe cases, it can result in foot ulcers, and eventually, limb amputation. Therefore, early and effective treatment of DPN is of great clinical importance (3). Currently, the treatment options for DPN are limited, with the main goals being symptom relief, improvement of nerve function, and prevention of foot ulceration (3). Dellon was among the first to discover that peripheral nerves become more sensitive to compression during diabetes and that the symptoms of DPN are caused by nerve compression (4). He pioneered the use of peripheral nerve decompression microsurgery for treating DPN, and clinical studies have confirmed that ND effectively alleviates nerve compression symptoms, improves nerve conduction velocity, relieves pain, and reduces the incidence of foot ulcers (5–16).

However, traditional ND surgery has certain limitations. The procedure typically requires extensive release of the tendon tissues around the nerve and the nerve epineurium. This not only potentially causes significant trauma to the patient but also may lead to postoperative scarring and fibrosis, which can result in new nerve compression and affect nerve function recovery (9, 17–21). Furthermore, releasing the nerve epineurium may impair the nerve’s blood supply, causing postoperative hypersensitivity, pain, and other adverse reactions (22–26). Diabetes patients often experience delayed wound healing, making it crucial to address the challenge of minimizing surgical trauma while improving therapeutic outcomes.

To address the limitations of traditional decompression, we developed a modified ND that performs targeted release at entrapment sites while avoiding unnecessary epineurial dissection, aiming to reduce surgical burden and preserve perineural vascularity (27–29). Although ND can alleviate symptoms in DPN patients, it does not address the underlying metabolic neuropathy caused by diabetes. Research has shown that chronic hyperglycemia leads to a reduction in the synthesis of nerve growth factor (NGF) in DPN patients, which exacerbates nerve damage (30–32). As a key neurotrophin, NGF not only relieves nerve pain but also improves nerve conduction velocity and promotes nerve regeneration. Accordingly, exogenous NGF supplementation has been reported to improve nerve function in DPN (33–35).

Thus, this study aims to evaluate the efficacy and safety of Modified ND surgery, as well as the combination of Modified ND and murine nerve growth factor (MNGF) injection in the treatment of lower limb DPN, exploring new therapeutic strategies for DPN patients.

## 2 Materials and methods

### 2.1 Study design

This assessor-blinded, three-arm exploratory randomized controlled trial enrolled 42 patients with diabetic peripheral neuropathy (DPN) treated at the Department of Burns and Plastic Surgery, Zunyi Medical University, between September 2019 and February 2022. Informed consent was obtained from all participants prior to enrollment. The study was conducted in accordance with the Declaration of Helsinki, and the research protocol was registered with the Chinese Clinical Trial Registry (ChiCTR2100044552, ChiCTR2200060034) and approved by the hospital’s ethics committee. Inclusion criteria were: (1) diagnosis of type 2 diabetes mellitus; (2) presence of typical lower limb DPN symptoms, with exclusion of other neuropathies; (3) clinical and electrophysiological diagnosis of DPN; (4) failure of non-surgical treatment and willingness to undergo surgical intervention. Exclusion criteria included: (1) neuropathy caused by other conditions, such as cervical or lumbar spine diseases, cerebrovascular accidents, Guillain-Barré syndrome, or severe vascular diseases; (2) severe comorbidities, such as heart disease, renal failure, or active infections; (3) allergy to nerve growth factor treatment.

### 2.2 Randomization and blinding

Participants were randomized 1:1:1 using a computer-generated sequence prepared by an independent investigator. Allocation was concealed with sequentially numbered, opaque, sealed envelopes, opened only after eligibility confirmation and baseline assessments. Site investigators enrolled participants, and a study coordinator (not involved in assessments) assigned interventions by opening the next envelope. Outcome assessors and the data analyst were blinded to group allocation; surgeons and participants were not due to the nature of surgery.

### 2.3 Human lower limb peripheral nerve anatomy

The anatomical dissection study conducted by the Anatomical Research Department of Zunyi Medical University focused on the course of the major peripheral nerves of the lower limb and identified key anatomical sites of compression for the common peroneal nerve, tibial nerve, and deep peroneal nerve.

### 2.4 Interventions

**Figure 1:**
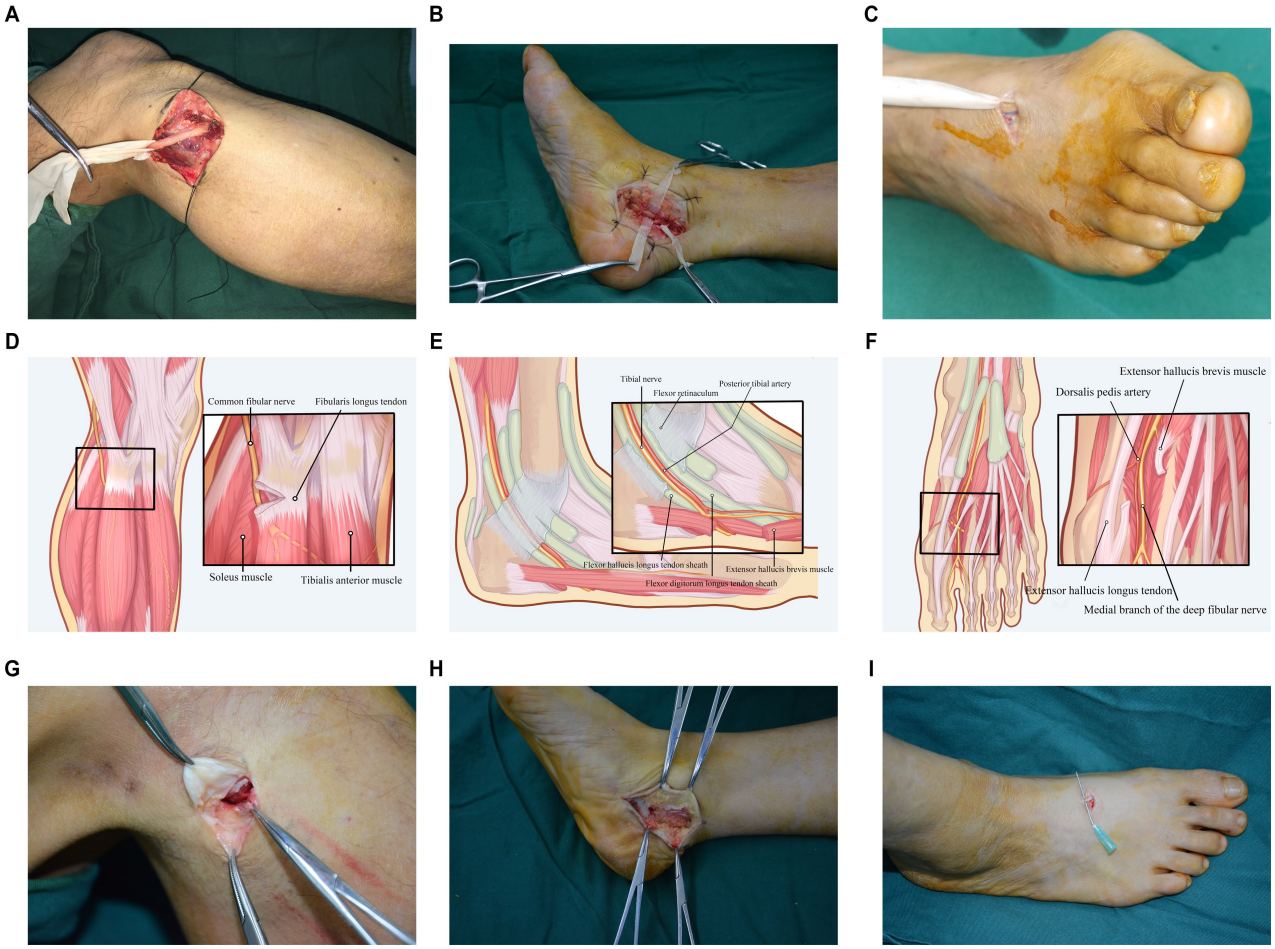
Intraoperative view of nerve decompression surgery. **(a)** Traditional common peroneal nerve decompression. **(b)** Traditional tibial nerve decompression. **(c)** Traditional deep peroneal nerve decompression. **(d)** Schematic diagram of modified common peroneal nerve decompression. **(e)** Schematic diagram of modified tibial nerve decompression. **(f)** Schematic diagram of modified deep peroneal nerve decompression. **(g)** Intraoperative view of modified common peroneal nerve decompression. **(h)** Intraoperative view of modified tibial nerve decompression. **(i)** Intraoperative view of modified deep peroneal nerve decompression.

Participants were randomly assigned to one of the following three treatment groups: *Traditional Nerve Decompression Group*: Common Peroneal Nerve Decompression (Figure 1a): A 5 cm long “S” shaped incision is made below the fibular head on both sides. After the incision is made layer by layer, the distribution of blood vessels and nerves is observed. The site of compression of the common peroneal nerve is identified. Tendon-like structures formed by the tendon, fascia, and ligaments at the origin of the fibularis longus muscle are severed. This process requires complete decompression of the nerve compression ring, with thorough separation and release of the nerve. Finally, under a microscope, the nerve epineurium is cut and decompressed to ensure effective alleviation of the nerve compression. Tibial Nerve Decompression (Figure 1b): A 5 cm long arcuate incision is made below the medial malleolus. After the incision is made layer by layer, the distribution of blood vessels and nerves is examined. The site of compression of the tibial nerve is identified. The flexor retinaculum in the tarsal tunnel, as well as the abductor hallucis muscle and surrounding connective tissues, are severed. This process requires complete decompression of the nerve compression ring, with full separation and release of the tibial nerve trunk and its branches. Under the microscope, the nerve epineurium is then cut and decompressed to ensure the tibial nerve is effectively decompressed. Deep Peroneal Nerve Decompression (Figure 1c): A 5 cm long “S” shaped incision is made between the first and second metatarsal bones on the dorsal aspect of the foot. After the incision is made layer by layer, the distribution of blood vessels and nerves is examined. The site of compression of the deep peroneal nerve is identified. Tendons of the extensor hallucis brevis muscle and surrounding connective tissues are severed. This process requires complete decompression of the nerve compression ring, with full separation and release of the deep peroneal nerve. Finally, under a microscope, the nerve epineurium is cut and decompressed to ensure the deep peroneal nerve is completely decompressed.

*Modified nerve decompression group*: Common Peroneal Nerve Decompression (Figures 1d,g): A 3 cm long “S” shaped incision is made below the fibular head on both sides. After making the incision layer by layer, the distribution of blood vessels and nerves is observed. The site of compression of the common peroneal nerve is identified. Tendon-like structures formed by the tendon, fascia, and ligaments at the origin of the fibularis longus muscle are severed. This process requires complete decompression of the nerve compression ring, but the nerve is not dissected. Tibial Nerve Decompression (Figures 1e,h): A 4 cm long arcuate incision is made below the medial malleolus. After making the incision layer by layer, the distribution of blood vessels and nerves is examined. The site of compression of the tibial nerve is identified. The flexor retinaculum in the tarsal tunnel, as well as the abductor hallucis muscle and surrounding connective tissues, are severed. This process requires complete decompression of the nerve compression ring, but the nerve is not dissected. Deep Peroneal Nerve Decompression (Figures 1f,i): A 2 cm long “S” shaped incision is made between the first and second metatarsal bones on the dorsal aspect of the foot. After making the incision layer by layer, the distribution of blood vessels and nerves is examined. The site of compression of the deep peroneal nerve is identified. Tendons of the extensor hallucis brevis muscle and surrounding connective tissues are severed. This process requires complete decompression of the nerve compression ring, but the nerve is not dissected.

*Modified nerve decompression + MNGF group*: The surgical procedure followed the same steps as described for the Modified ND Group. After meticulous hemostasis, MNGF (Shandong Weiming Bio-Medical Co., Ltd., National Drug Approval Number S20060052, specification 18 μg/vial) was reconstituted in 2 mL normal saline, and 18 μg was injected perineurally at the decompression site. The incision was then closed and dressed. Beginning postoperative day 1, 18 μg MNGF (18 μg in 2 mL saline) was administered intramuscularly once daily for 14 days.

### 2.5 Clinical assessments

All patients underwent the following assessments both before treatment and at 4 and 12 weeks post-treatment: 1. MNCV: Standard neurophysiological techniques were used to measure the motor nerve conduction velocities of the common peroneal nerve, tibial nerve, and deep peroneal nerve. 2. TCSS: All patients were assessed by the same investigator, who was blinded to the group assignments. The TCSS evaluates symptoms, lower limb reflexes (ankle and knee reflexes), and sensory testing. The total score ranges from 0 to 19, with a score ≥6 indicating the presence of DPN Scores of 6–8, 9–11, and 12–19 correspond to mild, moderate, and severe DPN, respectively. 3. VAS: Pain intensity was assessed using a 10 cm unmarked ruler, where 0 represents no pain and 10 represents the most severe pain. 4. 2-PD: The investigator used a blunt caliper to gradually decrease the distance between two points applied to the patient’s foot. The shortest distance at which the patient could distinguish between the two points was recorded. 5.10 g Monofilament Test: The investigator applied a 10 g monofilament to three areas of the sole of the foot and asked the patient whether they could feel the pressure. The test was repeated three times at each site to determine the presence of protective sensation. 6. Nerve CSA: The cross-sectional area of the common peroneal nerve at the fibular head was measured using ultrasound. The measurements were performed by the same investigator who was blinded to the group assignments. 7. Adverse Events: Adverse events occurring during the treatment period, including allergic reactions and infections, were recorded.

### 2.6 Statistical analysis

Statistical analyses were performed using SPSS 20.0. Continuous variables are presented as the mean ± standard deviation (𝑥 ± s). For comparisons between two groups, an independent *t*-test was used. For comparisons among multiple groups, one-way analysis of variance (ANOVA) was employed. If repeated measurements were taken (≥3 measurements), repeated measures ANOVA was used. Categorical data are presented as rates (n%), and the Chi-square test was used for comparisons. A *p*-value of < 0.05 was considered statistically significant.

## 3 Results

### 3.1 Human lower limb peripheral nerve anatomy

**Figure 2:**
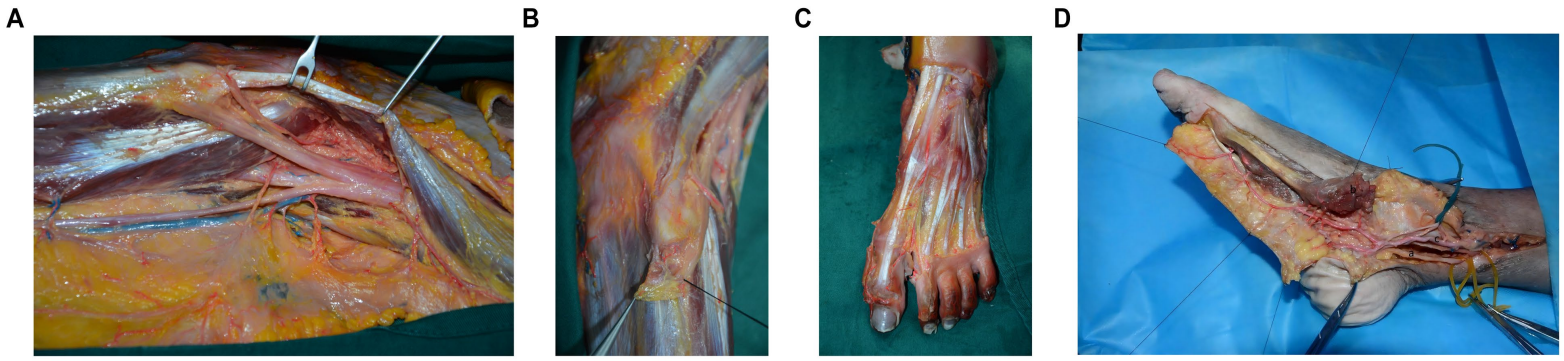
Anatomical Overview of the Major Lower Limb Nerves and Their Decompression. **(a)** Local anatomy of the common peroneal nerve. a: common peroneal nerve; b: lateral sural cutaneous nerve; c: fibular head; d: peroneus longus muscle; e: microvessels on the surface of the common peroneal nerve. **(b)** Decompression of the common peroneal nerve. a: common peroneal nerve; b: transected peroneus longus muscle. **(c)** Regional anatomy of the deep peroneal nerve. a: deep peroneal nerve; b: extensor hallucis brevis muscle; c: extensor hallucis longus muscle. **(d)** Regional anatomy of the tibial nerve. a: tibial nerve; b: severed tendon of the abductor hallucis muscle; c: posterior tibial artery.

Through lower limb anatomical dissection, the course of the three major affected nerves and their potential compression sites were identified. Compression of the common peroneal nerve is most commonly observed below the fibular head (Figure 2a,b), tibial nerve compression typically occurs in the tarsal tunnel region (Figure 2c), while the deep peroneal nerve is prone to compression between the first and second metatarsals (Figure 2d). These anatomical constriction sites provide clear surgical targets, facilitating precise localization and surgical intervention in nerve decompression procedures, thereby effectively minimizing unnecessary damage.

### 3.2 Baseline characteristics

A total of 42 patients were included in this study, divided into three groups: the Traditional ND group (Group 1, *n* = 12), the Modified ND group (Group 2, *n* = 16), and the Modified ND + MNGF group (Group 3, *n* = 14). There were no significant differences in age, gender, duration of diabetes, HbA1c levels, and the severity of DPN among the three groups (*p* > 0.05), indicating that the baseline characteristics of the groups were similar (Table 1).

**Table 1:** Baseline characteristics of patients across treatment groups.

Characteristic	Traditional ND	Modified ND	Modified ND + MNGF	*p*-value
Cases (n)	12	16	14	
Gender (M/F)	5/7	10/6	7/7	0.538
Age (years)	53.8 ± 11.4	59.5 ± 7.6	55.3 ± 10.7	1.189
BMI (kg/m^2^)	23.2 ± 1.4	24.2 ± 1.3	23.5 ± 1.4	0.085
Diabetes duration (years)	10.0 ± 4.1	10.6 ± 4.9	10.1 ± 6.8	0.971
Lower limb pain duration (months)	2.1 ± 1.3	2.4 ± 1.1	2.4 ± 1.4	0.923
TCSS score (pre-op)	14.8 ± 2.9	14.3 ± 2.7	14.1 ± 2.8	0.988
VAS score (pre-op)	7.3 ± 1.7	6.6 ± 1.9	6.3 ± 2.0	0.952
2-PD (pre-op, mm)	32.5 ± 5.8	30.5 ± 5.9	32.9 ± 4.2	0.379
10 g Monofilament (pre-op, %)	22(91.7%)	26(81.3%)	23(82.1%)	0.517

### 3.3 Motor nerve conduction velocity

**Figure 3:**
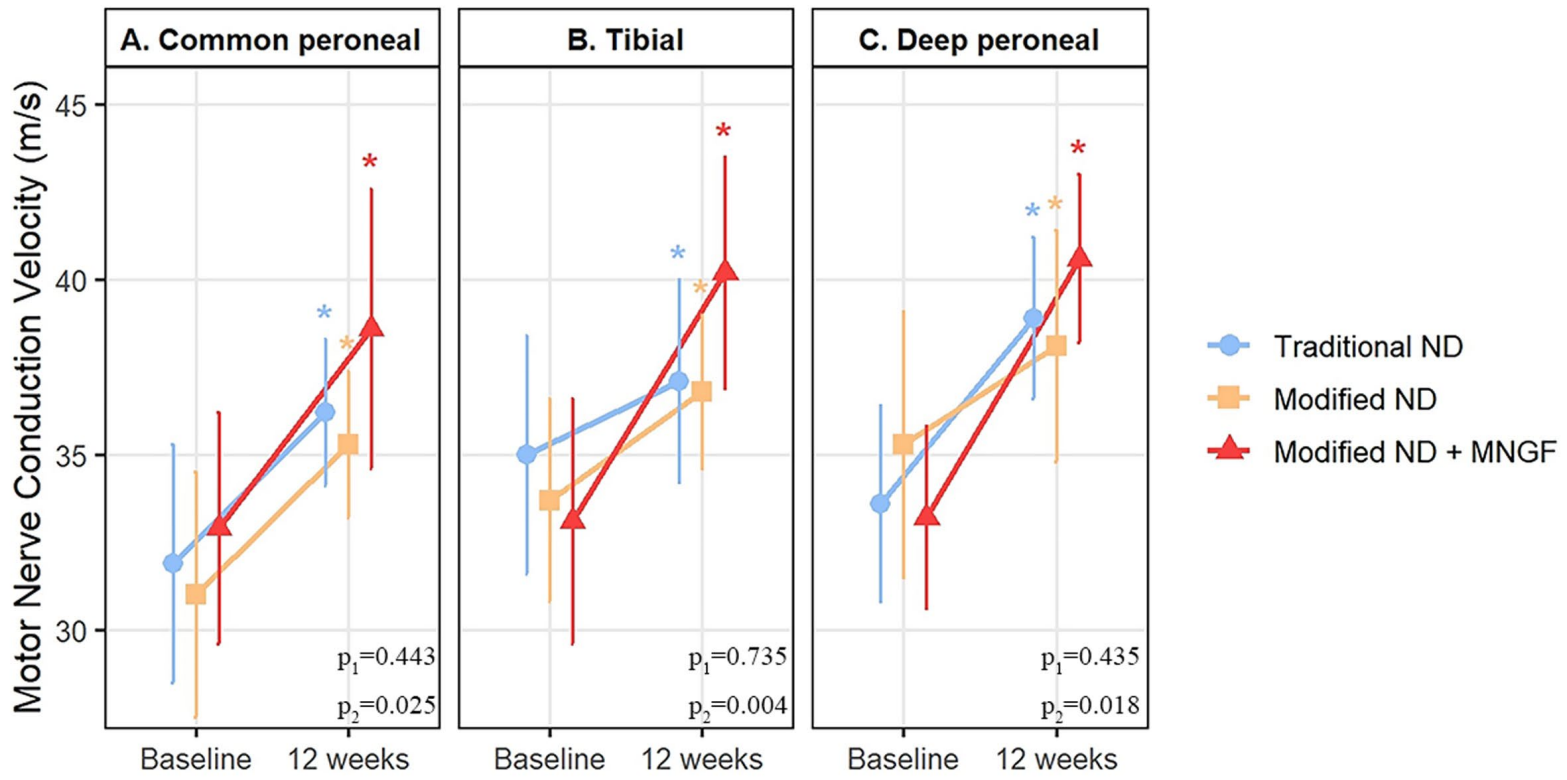
Comparison of motor nerve conduction velocity (MNCV) in **(A)** common peroneal, (B) tibial, and (C) deep peroneal nerves between the modified ND, traditional ND, and modified ND + MNGF groups. Data are mean ± SD at 0, 4, and 12 weeks. * indicates a significant within-group change from baseline at that time point (*p* < 0.05). P1, Modified ND vs. Traditional ND (post-treatment); P2, Modified ND + MNGF vs. Traditional ND (post-treatment).

There were no significant differences in MNCV between the groups before treatment. Twelve weeks post-treatment, MNCV significantly increased in all treatment groups compared to pre-treatment values (*p* < 0.05). Specifically, in the Traditional ND group, the common peroneal nerve MNCV increased from 31.9 ± 3.4 m/s preoperatively to 36.2 ± 2.1 m/s postoperatively (*p* < 0.01). In the Modified ND group, the common peroneal nerve MNCV increased from 31.0 ± 3.5 m/s to 35.3 ± 2.1 m/s (*p* < 0.01). In the Modified ND + MNGF group, the common peroneal nerve MNCV increased from 32.9 ± 3.3 m/s to 38.6 ± 4.0 m/s (*p* < 0.01). There was no significant difference between the Modified ND group and the Traditional ND group post-treatment (*p* > 0.05), while the Modified ND + MNGF group showed a significant improvement in MNCV compared to the Traditional ND group (*p* < 0.05) (Figure 3).

### 3.4 TCSS

**Figure 4:**
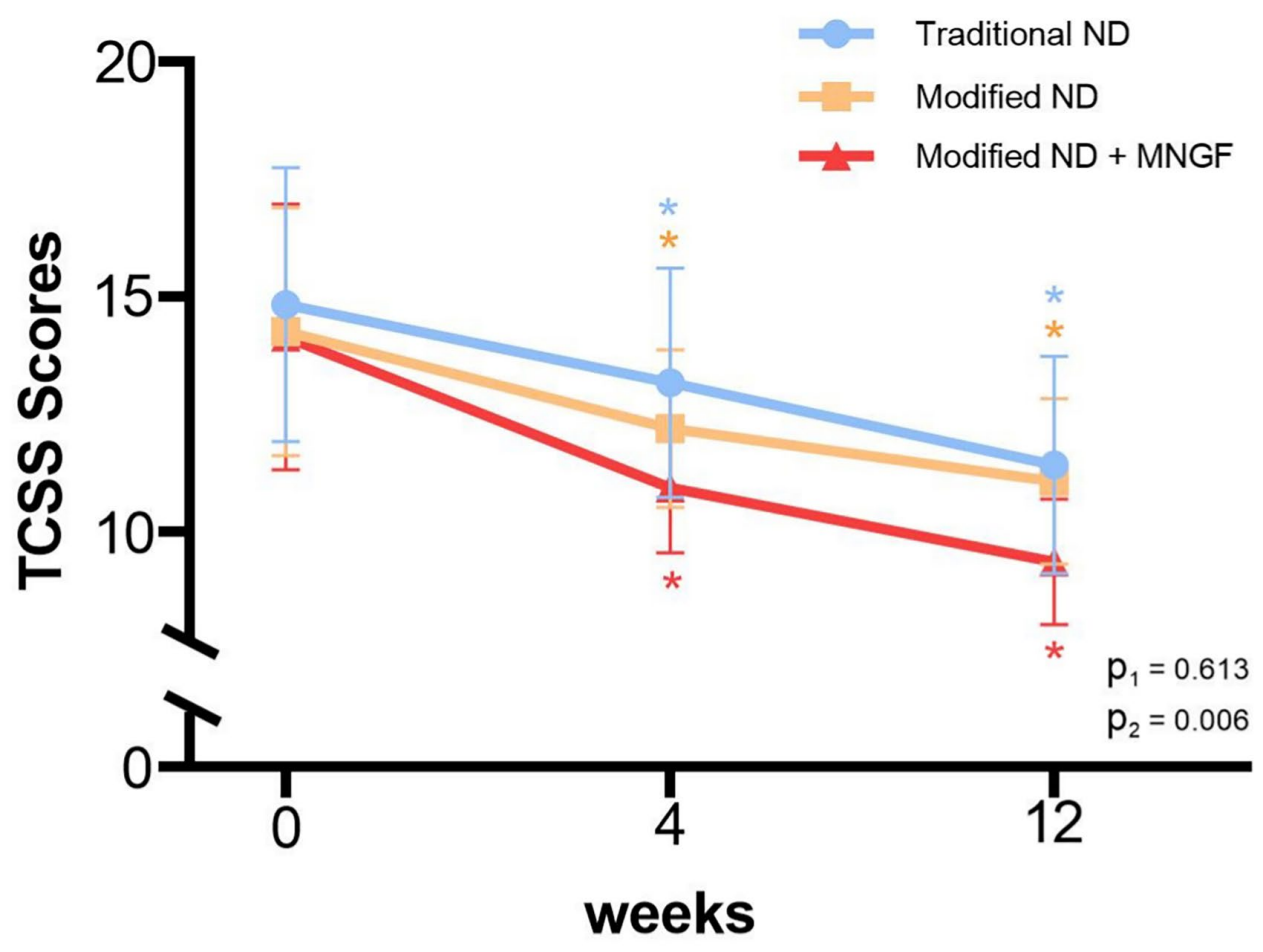
Comparison of TCSS in the modified ND, traditional ND, and modified ND + MNGF groups. Data are mean ± SD at 0, 4, and 12 weeks. * indicates a significant within-group change from baseline at that time point (*p* < 0.05). P1, Modified ND vs. Traditional ND (post-treatment); P2, Modified ND + MNGF vs. Traditional ND (post-treatment).

The TCSS scores, reflecting the overall clinical manifestations of DPN, showed no significant differences between the groups before treatment, but significant improvement was observed in all groups post-treatment (*p* < 0.05). Specifically, in the Traditional ND group, TCSS decreased from 14.8 ± 2.9 preoperatively to 11.4 ± 2.3 postoperatively (*p* < 0.01). In the Modified ND group, TCSS decreased from 14.3 ± 2.7 to 11.1 ± 1.8 (p < 0.01). In the Modified ND + MNGF group, TCSS decreased from 14.1 ± 2.8 to 10.9 ± 1.4 (*p* < 0.01). There was no significant difference between the Modified ND group and the Traditional ND group post-treatment (*p* > 0.05), while the Modified ND + MNGF group showed a significant improvement in TCSS compared to the Traditional ND group (*p* < 0.05) (Figure 4).

### 3.5 2-PD

There were no significant differences in 2-PD scores between the groups before treatment. Post-treatment, all groups showed significant sensory recovery compared to pre-treatment values. Specifically, in the Traditional ND group, 2-PD improved from 32.5 ± 5.8 mm preoperatively to 24.6 ± 3.3 mm postoperatively (*p* < 0.01). In the Modified ND group, 2-PD improved from 30.5 ± 5.9 mm to 24.0 ± 4.1 mm (*p* < 0.01). In the Modified ND + MNGF group, 2-PD improved from 32.9 ± 4.2 mm to 21.3 ± 2.3 mm (*p* < 0.01). There was no significant difference between the Modified ND group and the Traditional ND group post-treatment (*p* > 0.05), while the Modified ND + MNGF group showed a significant improvement in 2-PD compared to the Traditional ND group (*p* < 0.05) (Table 2).

**Table 2:** Comparison of 2-PD scores in the modified ND, traditional ND, and modified ND + MNGF groups.

Group	Pre-treatment	Post-treatment 30 days	Post-treatment 90 days
Traditional ND	32.5 ± 5.8	27.8 ± 3.4	24.6 ± 3.3
Modified ND	30.5 ± 5.9	26.3 ± 4.3	24.0 ± 4.1
Modified ND + MNGF	32.9 ± 4.2	23.3 ± 2.4	21.3 ± 2.3
P-value		P1 = 0.267; P2 = 0.002	P1 = 0.651; P2 = 0.017

P1 represents the comparison between the modified ND group and the traditional ND group post-treatment; P2 represents the comparison between the Modified ND + MNGF group and the traditional ND group post-treatment.

### 3.6 10 g monofilament test

There were no significant differences between the groups before treatment. However, at 30 days and 90 days post-treatment, all three groups showed significant improvement compared to pre-treatment values (*p* < 0.05). Notably, the Modified ND + MNGF group demonstrated a significantly greater improvement post-treatment compared to the Traditional ND group (*p* < 0.05) (Table 3).

**Table 3:** Comparison of 10 g monofilament test results between modified ND, traditional ND, and modified ND + MNGF groups.

Group	Pre-treatment		Post-treatment 12 weeks	
	Positive	Negative	Positive	Negative
Traditional ND	22 (91.7)	2 (8.3)	11 (45.8)	13 (54.2)
Modified ND	26 (81.3)	6 (18.8)	10 (31.3)	22 (68.8)
Modified ND + MNGF	23 (82.1)	5 (17.9)	2 (7.1)	26 (92.9)
P-value				P1 = 0.282; P2 = 0.000

P1 represents the comparison between the modified ND group and the traditional ND group post-treatment; P2 represents the comparison between the Modified ND + MNGF group and the traditional ND group post-treatment.

### 3.7 VAS

There were no significant differences in VAS scores between the groups before treatment. At 4 and 12 weeks post-treatment, VAS scores significantly decreased in all groups compared to pre-treatment values (*p* < 0.05). Specifically, in the Traditional ND group, VAS decreased from 7.3 ± 1.7 preoperatively to 3.7 ± 0.8 postoperatively (*p* < 0.01). In the Modified ND group, VAS decreased from 6.6 ± 1.9 to 3.1 ± 0.7 (*p* < 0.01). In the Modified ND + MNGF group, VAS decreased from 6.3 ± 2.0 to 3.1 ± 0.9 (*p* < 0.01). There was no significant difference between the Modified ND group and the Traditional ND group post-treatment (*p* > 0.05), while the Modified ND + MNGF group showed a greater reduction in VAS compared to the Traditional ND group (*p* < 0.05) (Table 4).

**Table 4:** Comparison of VAS Scores in the modified ND, traditional ND, and modified ND + MNGF groups.

Group	Pre-treatment	Post-treatment 30 days	Post-treatment 90 days
Traditional ND	7.3 ± 1.7	5.2 ± 1.0	3.7 ± 0.8
Modified ND	6.6 ± 1.9	4.7 ± 0.7	3.1 ± 0.7
Modified ND + MNGF	6.3 ± 2.0	4.8 ± 0.8	3.1 ± 0.9
*p*-value		P1 = 0.132; P2 = 0.167	P1 = 0.079; P2 = 0.098

P1 represents the comparison between the modified ND group and the traditional ND group post-treatment; P2 represents the comparison between the Modified ND + MNGF group and the traditional ND group post-treatment.

### 3.8 Nerve cross-sectional area

There were no significant differences in common peroneal nerve CSA between the groups before treatment. Post-treatment, all three groups showed a significant reduction in common peroneal nerve CSA compared to pre-treatment values (*p* < 0.05). However, there were no significant differences between the groups post-treatment (Table 5).

**Table 5:** Comparison of common peroneal nerve CSA in the modified ND, traditional ND, and modified ND + MNGF groups.

Group	Pre-treatment	Post-treatment
Traditional ND	12.3 ± 2.3	11.1 ± 1.5
Modified ND	11.9 ± 2.8	10.6 ± 1.3
Modified ND + MNGF	11.9 ± 2.3	9.9 ± 1.6
*P*-value		P1 = 0.358; p2 = 0.052

P1 represents the comparison between the Modified ND group and the traditional ND group post-treatment; P2 represents the comparison between the modified ND + MNGF group and the traditional ND group post-treatment.

### 3.9 Adverse events

In the Traditional ND group, three patients experienced increased hypersensitivity with elevated VAS scores during postoperative follow-up. Each group had one patient with delayed wound healing, which resolved with enhanced dressing changes. No cases of surgical site infection, new ulcers, or amputations were reported.

## 4 Discussion

This study evaluated the efficacy and safety of modified ND and the combination of modified ND with MNGF injection in the treatment of lower limb DPN. The results indicate that Traditional ND, Modified ND, and Modified ND + MNGF significantly improved nerve conduction velocity, pain intensity, and sensory function in DPN patients. Compared to traditional nerve decompression, the modified ND technique reduced complications and trauma by avoiding excessive dissection of the nerve epineurium, while achieving similar therapeutic outcomes. Furthermore, the Modified ND + MNGF group demonstrated superior postoperative outcomes compared to the Traditional ND group.

According to the “dual compression” hypothesis, the pathophysiological mechanisms of DPN involve two primary compressive factors: Firstly, oxidative stress and the accumulation of advanced glycation end-products (AGEs) induced by prolonged hyperglycemia disrupt normal nerve metabolism, leading to nerve edema and reduced blood supply, which in turn results in functional degeneration. Secondly, the accumulation of AGEs leads to nerve sclerosis, decreased elasticity, and impaired gliding ability. Additionally, the thickening and edema of fibrous connective tissues at physiological anatomical constriction sites further exacerbate chronic nerve compression, thereby promoting the onset and progression of DPN (4, 6, 11, 36).

Studies have shown that, compared with healthy individuals, patients with DPN have a significantly reduced intraneural vascular network. Any intraoperative injury to the surrounding vessels may further compromise neural perfusion; therefore, preserving the vascular supply is crucial during decompression (37). Anatomical observations revealed that the common peroneal nerve is most vulnerable to compression as it curves around the fibular head before entering the fibularis longus muscle, whereas the segment located in the superficial fascial layer proximal to this point is relatively “safe.” Accordingly, decompression is achieved by incising the tendinous tissue at the origin of the fibularis longus to release the common peroneal nerve. The deep peroneal nerve is primarily compressed by the extensor hallucis brevis tendon; thus, sectioning this tendon effectively relieves the entrapment. The tibial nerve, positioned at the deepest portion of the tarsal tunnel, is decompressed by incising the tendinous tissue over the tarsal tunnel and releasing the surrounding tissues at the entry points of the medial and lateral plantar nerves, while avoiding excessive longitudinal dissection to minimize ischemic risk. This modified ND technique maximizes protection of the nerve trunk and its vascular supply while shortening operative time, reducing intraoperative bleeding, and lowering postoperative complications. Compared with traditional ND, it achieved comparable improvements in nerve conduction velocity, clinical scores, and symptom relief, with smoother recovery and greater surgical safety (22, 23).

Chronic hyperglycemia is associated with reductions in NGF and its receptors, correlating inversely with DPN severity (38, 39). As a well-characterized neurotrophin, NGF supports neuronal survival, Schwann-cell vitality, and angiogenesis, and has been shown to promote peripheral nerve regeneration (40). Murine NGF, which is highly homologous to human NGF, has been reported to lower TCSS scores, improve nerve conduction velocity, and enhance lower-limb sensory function in DPN (41). In our trial, modified ND plus MNGF was associated with additional early improvements versus surgery alone, suggesting a complementary, biologically plausible adjunct for DPN management.

The strength of this study lies in its innovative approach, proposing a more minimally invasive ND surgery for the treatment of DPN, and combining Modified ND surgery with MNGF, providing a novel therapeutic strategy for DPN. Through multi-dimensional evaluation, we thoroughly assessed the efficacy of the modified technique. However, this study has limitations: The sample size was relatively small and recruitment occurred at a single center, which may limit generalizability. Sensory outcomes were assessed with static two-point discrimination (s2PD/Weber) and a 10-g monofilament only; omission of moving two-point discrimination (m2PD/Dellon) and pressure-specified or broader quantitative sensory testing may have underdetected early or subtle recovery. The 12-week follow-up is short for DPN. Despite signals of benefit from existing studies, high-quality randomized evidence for ND in DPN remains limited. Consistent with the 2025 ADA Standards of Care, team-based, integrated management remains the priority in DPN/diabetic-foot care (optimized glycemic and cardiometabolic control, biomechanical off-loading and foot care, vascular assessment with revascularization when indicated, and patient education) (42). Within this framework, ND should be evaluated as a potential adjunct for carefully selected patients with clinical evidence of entrapment rather than as a stand-alone solution. Future studies should be adequately powered, multicenter, and longer-term, and adopt standardized, patient-centered outcomes (e.g., ulcer incidence or time-to-ulcer, pain/function, quality of life).

## Data Availability

The original contributions presented in the study are included in the article/supplementary material, further inquiries can be directed to the corresponding authors.
